# Community-Wide Monitoring of Lead in Drinking Water Distribution Systems Using Hand-Held Voltammetric Sensors and Geographic Information Systems

**DOI:** 10.1021/acsomega.5c01580

**Published:** 2025-05-01

**Authors:** Yigit C. Bozkurt, Al-Monsur Jiaul Haque, Connor Sullivan, Boyang Xiang, Yidong Zhu, Mohammad Arif Ul Alam, Pradeep U. Kurup

**Affiliations:** † Department of Civil and Environmental Engineering, University of Massachusetts Lowell, Lowell, Massachusetts 01854, United States; ‡ Department of Miner School of Computer & Information Sciences, University of Massachusetts Lowell, Lowell, Massachusetts 01854, United States

## Abstract

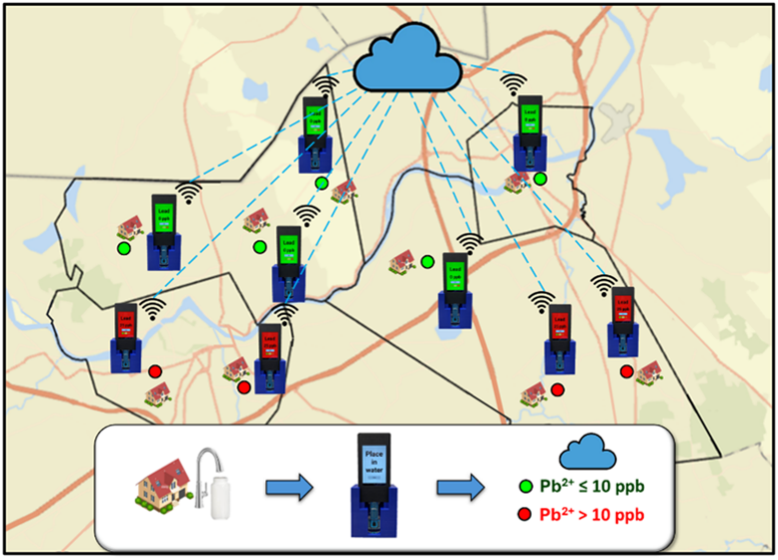

Lead (Pb^2+^) contamination in drinking water remains a critical public health concern, particularly for children, due to lead pipes and plumbing in many water systems. Conventional Pb^2+^ detection methods, such as ICP-MS and AAS, are costly, time-intensive, and require specialized personnel. In this study, we developed and utilized a portable voltammetric Pb^2+^ detection system, the E-Tongue, which features a mercury-free, gold nanostar-modified screen-printed carbon electrode, and nontoxic buffer reagents (0.1 M sodium acetate, 0.1 mM potassium ferrocyanide, pH 4.5). The E-Tongue provides Pb^2+^ detection within 5 min with a method detection limit of 1.6 ppb and a wide linear range of 5–200 ppb. Results demonstrated the E-Tongue’s ability to detect Pb^2+^ above the EPA action level (10 ppb), even in high Cu^2+^ conditions (up to 1.3 ppm), with Pb^2+^ recovery of 84–105% and RSD < 10%. The E-Tongue’s color-coded and quantitative feedback enables nonexperts to test tap water and share data, facilitating community-driven monitoring and intervention strategies. Additionally, spatial analysis revealed that Andover had the most alkaline and conductive tap water, while Lawrence exhibited neutral water on average. The E-Tongue empowers communities, demonstrating the potential of participatory approaches for lead detection and mitigation in water networks.

## 1 Introduction

Water is vital for supporting life, but its safety is not always guaranteed due to numerous sources of heavy metal contamination, even in developed countries.^1^ Heavy metals, such as lead (Pb^2+^) and copper (Cu^2+^) in drinking water have emerged as a critical concern, drawing attention to the significant risks they pose to public health.^2−5^ Particularly, Pb^2+^ contamination poses alarming health risks even at trace concentrations in the parts per billion (ppb) range. As a result, the US Environmental Protection Agency (EPA) established an Action Level of 15 ppb in the Lead and Copper Rule in 1991 and a Trigger Level of 10 ppb in the Lead and Copper Rule Revision (LCRR) in 2021. The Action Level was subsequently changed to 10 ppb under the Lead and Copper Rule Improvements (LCRI) in 2024, removing the Trigger Level.^6,7^ However, due to its nonbiodegradable nature and neurotoxicity, there is no safe level of lead in drinking water. Continuous exposure to lead negatively affects various human organs and impairs children’s mental development.^8−13^

Drinking water treatment plants routinely test to ensure treated water contains nondetectable concentrations of Pb^2+^. However, contamination remains a concern due to pipes in the drinking water distribution system and home plumbing materials, such as lead service lines, lead goosenecks, lead pigtails, galvanized pipes, copper pipes with lead solder, and fixtures.^14−16^ Testing at individual homes is performed, but regulations only require municipalities to test a small number of homes every 1 to 3 years, leaving many homes untested.^17^ This limitation restricts the number of samples tested by drinking water programs, necessitating the allocation of limited tests across all homes in a town. Pb^2+^ can be detected in tap water using EPA-approved standard methods.^18,19^ However, these methods have significant limitations: they require large laboratory-based instruments such as atomic absorption spectrometer (AAS) and inductively coupled plasma mass spectrometer (ICP-MS), highly trained operators, centralized certified laboratories, and have a reporting time of 1–2 weeks, including sample collection, mailing, and testing.^20,21^ Due to these limitations, electrochemical methods like voltammetry have gained attention as alternatives because they can be miniaturized for on-site monitoring, are cost-effective, provide results within minutes, and can be operated by nontechnical personnel.^20−25^

Numerous studies have reported voltammetric methods for detecting trace level Pb^2+^. However, existing voltammetric methods have limitations that prevent them from being practically applied for widespread drinking water testing.^26^ These methods usually require multiple modifications to be applied to screen-printed electrodes, making mass production of these sensors impractical.^27−29^ Additionally, these methods require nitrogen purging and/or stirring during preconcentration, which limits their use outside of a laboratory.^30−33^ Furthermore, the EPA has approved a voltammetric Pb^2+^ detection method, Method 1001: Lead in Drinking Water by Differential Pulse Anodic Stripping Voltammetry.^34^ However, this method uses toxic mercury electrodes. Generating mercury waste to detect the toxic heavy metal Pb^2+^ is not an environmentally sustainable solution. Moreover, this lead method (Palintest model SA1100) has been shown to provide false negatives due to complex water matrices in real tap water samples.^35^

**Figure 1:**
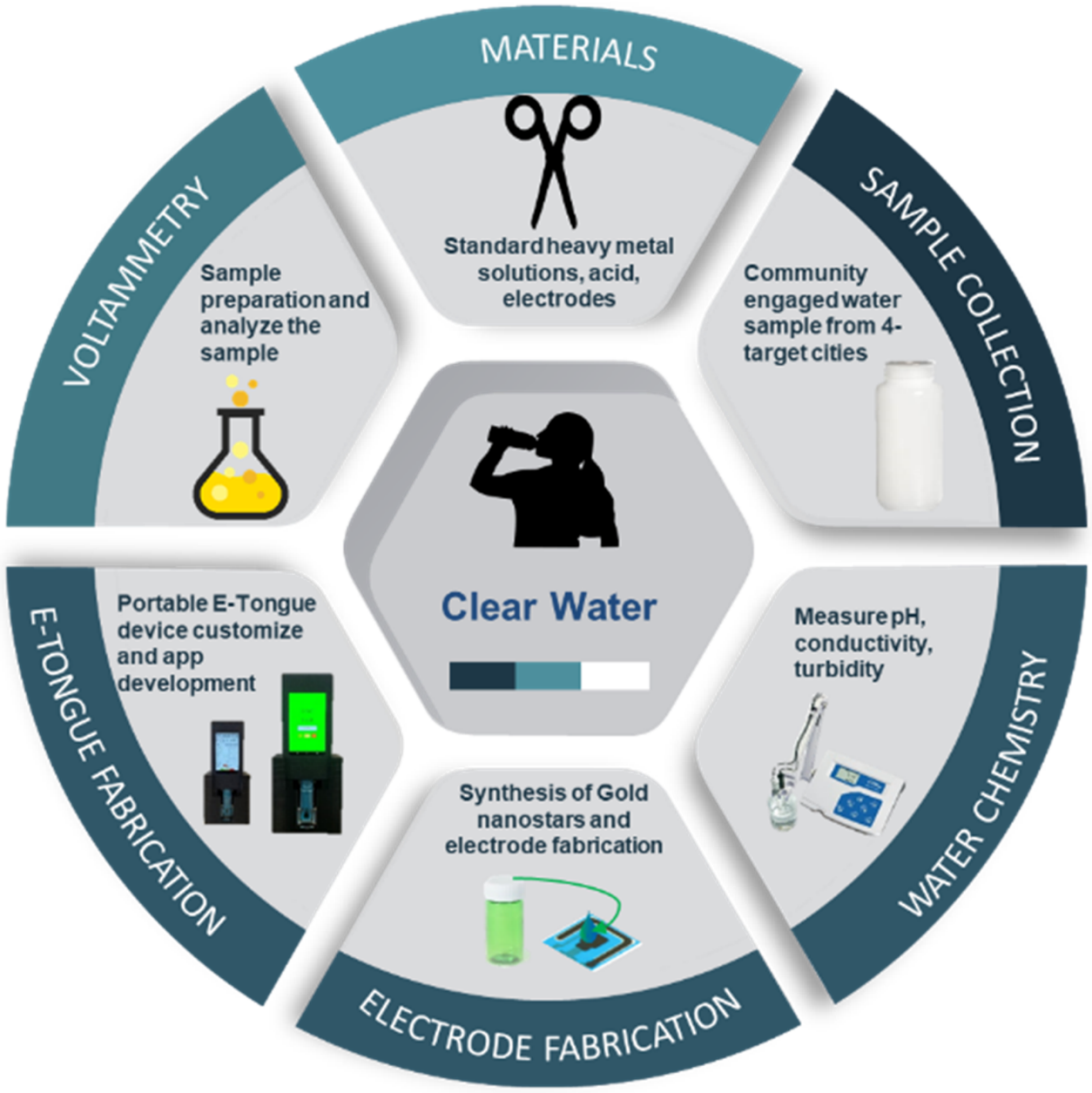
Schematic representation of the methodology for water quality monitoring using portable E-Tongue sensors. Key steps include material preparation, sample collection, water chemistry analysis, electrode fabrication, E-Tongue customization, and voltammetry analysis to ensure clear water monitoring.

To address these limitations, this study demonstrates the development of a voltammetric method for Pb^2+^ detection, the integration of this method into a hand-held device, the E-Tongue, and evaluation of the efficacy of the E-Tongue for community-wide monitoring of Pb^2+^ in drinking water distribution systems (Figure 1). The E-Tongue is a scalable, cost-effective solution for widespread Pb^2+^ monitoring. The device uses mercury-free sensors (i.e., gold nanostar) and nontoxic reagents, is user-friendly, and is designed for nonexperts. The gold nanostar-based sensor enables the device to achieve high sensitivity and low detection limits, taking advantage of the high electrocatalytic activity and the large active surface area of gold nanostars.^36,37^ Furthermore, we previously reported the use of gold nanostars for detecting other heavy metal ions with good sensitivity and reproducibility.^38,39^ The developed method can detect Pb^2+^ even in the presence of high levels of Cu^2+^. Android applications were also developed to provide color-coded results according to the Action Level of Pb^2+^. Additionally, water chemistry parameters are spatially analyzed using geographic information system (GIS) maps to identify trends in drinking water quality. This approach highlights the importance of in situ Pb^2+^ monitoring, benefiting public water suppliers, regulatory agencies, and the public.

## 2 Materials and Methods

### 2.1 Materials

Standard heavy metal solutions (1000 ppm Pb and Cu, AAS grade) were obtained from Fisher Scientific. Nitric acid (67%) was sourced from VWR. HEPES 2-[4-(2-hydroxyethyl)piperazin-1-yl] ethanesulfonic acid (99.5%), 3 M sodium acetate buffer solution, potassium hexacyanoferrate (II) trihydrate (98.5%), gold(III) chloride solution (30%), hydrochloric acid (37%), and sodium hydroxide (97%) were obtained from Sigma-Aldrich. All aqueous solutions were prepared using Milli-Q water (18.2 MΩ cm^–1^ at 25 °C) from a Barnstead Smart2PurePro water purification system (Thermo Scientific). Screen-printed carbon electrodes (SPCEs) were purchased from Pine Research. The electrode pattern includes a carbon working electrode (4 × 5 mm^2^), a carbon counter electrode, and a silver/silver chloride (Ag/AgCl) reference electrode.

### 2.2 Community Engaged Water Sample Collection

Tap water samples were collected by residents from four Massachusetts towns/cities: Andover, Dracut, Lawrence, and Lowell, MA. Residents collected the first-liter and the fifth-liter samples in 1 L high-density polyethylene plastic wide-mouth bottles after at least 6 h of stagnation.^40,41^ Samples were acidified below pH 2 by adding 2 mL of 70% nitric acid within 14 days of sampling. Prior to acidification, pH, conductivity, and turbidity were measured. Samples were then refrigerated at +4 °C temperature for at least 16 h before testing, allowing for six months of storage. The research team engaged residents through postcards, community outreach workshops, events, and guest lessons at local schools. This effort resulted in the collection of tap water samples from 317 homes. A total of 634 samples (i.e., 317 first and fifth liters) were collected between June 1, 2023, and August 28, 2024. Spatial analysis was conducted using Esri’s ArcGIS Pro on Windows-operated computers.

### 2.3 Measurement of Lead, Copper, and Water Chemistry Parameters

The pH, conductivity, and turbidity of each sample were measured using a Fisherbrand FE150 pH benchtop meter, OHAUS Aquasearcher a-AB33EC Conductivity Bench Meter, and VWR Turbidity Meter, respectively. Confirmatory lead and copper testing was performed using the PerkinElmer PinAAcle 900T Atomic Absorption Spectroscopy (AAS) or the Agilent 7900 Inductively Coupled Plasma-Mass Spectrometer (ICP-MS). Pb^2+^ was detected using either EPA Method 200.9 (Determination of Trace Elements by Stabilized Temperature Graphite Furnace AAS) or Method 200.8 (Determination of Trace Elements in Waters and Wastes by ICP-MS). Cu^2+^ was detected using either EPA Method 200.8 or the National Environmental Methods Index’s Standard Method 3111B (Metals in Water by Flame AAS). If Pb^2+^ or Cu^2+^ were detected above the EPA Action Level, the results were verified by a certified laboratory, Nashoba Analytical or Granite State Analytical Services. In addition, Nashoba Analytical tested for other water chemistry parameters, including calcium, magnesium, manganese, potassium, sodium, hardness, pH, chloride, fluoride, nitrate as N, and sulfate.

### 2.4 Synthesis of Gold Nanostars and Electrode Fabrication

All voltammetric experiments were performed with gold nanostars-modified screen-printed carbon electrodes (AuNS/SPCEs). SPCEs were obtained from Pine Research. AuNS was synthesized using HEPES, a reducing and capping agent. Detailed synthesis and characterizations of AuNS have been previously reported.^36−39,42−44^ Transmission electron microscopy images have shown clear spiked structures, with approximate average tip-to-tip diameter of 49 ± 14 nm, average spike length of 16 ± 1 nm, and average inner diameter of 23 ± 6 nm.^36^ The X-ray diffraction analysis of the AuNS powder showed four diffraction peaks at 38.3, 44.1, 64.7, and 77.3°, which are consistent with standard crystalline gold, with a predominant orientation plane of (111).^37^ Cyclic voltammetry experiments on AuNS/SPCE and bare SPCE, showed higher oxidation/reduction peak currents at AuNS/SPCE as well as smaller redox peak separation, indicating increased electroactive surface area and improved efficiency of the electron transfer process, respectively.^38^ Electrochemical impedance spectroscopy experiments demonstrated the charge transfer resistance (R_CT_) decreased significantly from 2.4 kΩ (bare SPCE) to 0.8 kΩ (AuNS/SPCE) indicating the faster heterogeneous electron transfer at the AuNS/SPCE.^37^ The authors attribute the improved detection of lead and other heavy metals, to the AuNS/SPCE greater surface area, improved efficiency of the electron transfer process, and reduced charge transfer resistance. During this study, UV Vis analysis (see Figure S1) was performed on all suspensions of AuNS, to ensure they were comparable with the suspensions used in the team’s prior work.

UV–visible spectrophotometric characterization of synthesized gold nanostar (AuNS) solutions was performed using a Hach UV–vis DR6000. The AuNS suspension was analyzed to check peak locations λ_1_ and λ_2_, corresponding to the spherical and spike portions of the AuNS, respectively (Figure S1). The suspension was then stored at +4 °C for up to three months for subsequent use in drop-casting. SPCEs were washed with DI water. Twenty μL of AuNS suspension was drop-cast onto the working electrode of the SPCE using an adjustable Eppendorf pipet. Finally, the AuNS/SPCEs were air-dried at room temperature under a WhisperFlow Fan Filter Unit for 24 h before use.

### 2.5 E-Tongue Fabrication and Voltammetric Measurements

The E-Tongue is a hand-held device consisting of a PalmSens’s EmStat Pico module as the potentiostat and an Android operating system integrated into a hand-held device. The E-Tongue Android applications were developed using an open-source example application from PalmSens’s software development kits. The E-Tongue was programmed with experimental parameters for the square wave stripping analysis of Pb^2+^. The AuNS/SPCEs were conditioned at 0.3 V vs Ag/AgCl for 10 s. Then, a deposition potential of −0.9 V vs Ag/AgCl was applied for 300 s. Finally, the potential was scanned from −0.9 to 0 V vs Ag/AgCl in a square waveform with parameters of 30 mV amplitude, 10 mV step increment, and 50 Hz (s^–1^) frequency following the deposition. All calibration samples and presented tap water samples were analyzed in triplicate.

All samples were prepared by adding 1 mL of 1 M sodium acetate buffer solution (pH 4.5) containing 1 mM potassium ferrocyanide. The buffer solution was prepared by diluting 10 mL of 3 M sodium acetate buffer with deionized water (DI) and adjusting the pH by adding ∼0.6 mL of concentrated hydrochloric acid. Next, 3 mL of 10 mM potassium ferrocyanide was added to the diluted sodium acetate buffer solution and the final volume was brought to 30 mL. The sodium acetate buffer solution was freshly prepared before the Pb^2+^ detection experiment. To analyze tap water samples, 1 mL of the buffer solution was added to 9 mL of tap water sample in a 20 mL glass vial used as the electrochemical cell. This resulted in 0.1 M sodium acetate buffer electrolyte and 0.1 mM potassium ferrocyanide as their final concentrations. No stirring or nitrogen purging was used during the voltammetric experiment, as it was shown to not enhance peak height or otherwise change the voltammograms (Figure S5)

## 3 Results and Discussion

### 3.1 Measurement of pH, Conductivity, and Turbidity of Tap Water Samples

**Figure 2:**
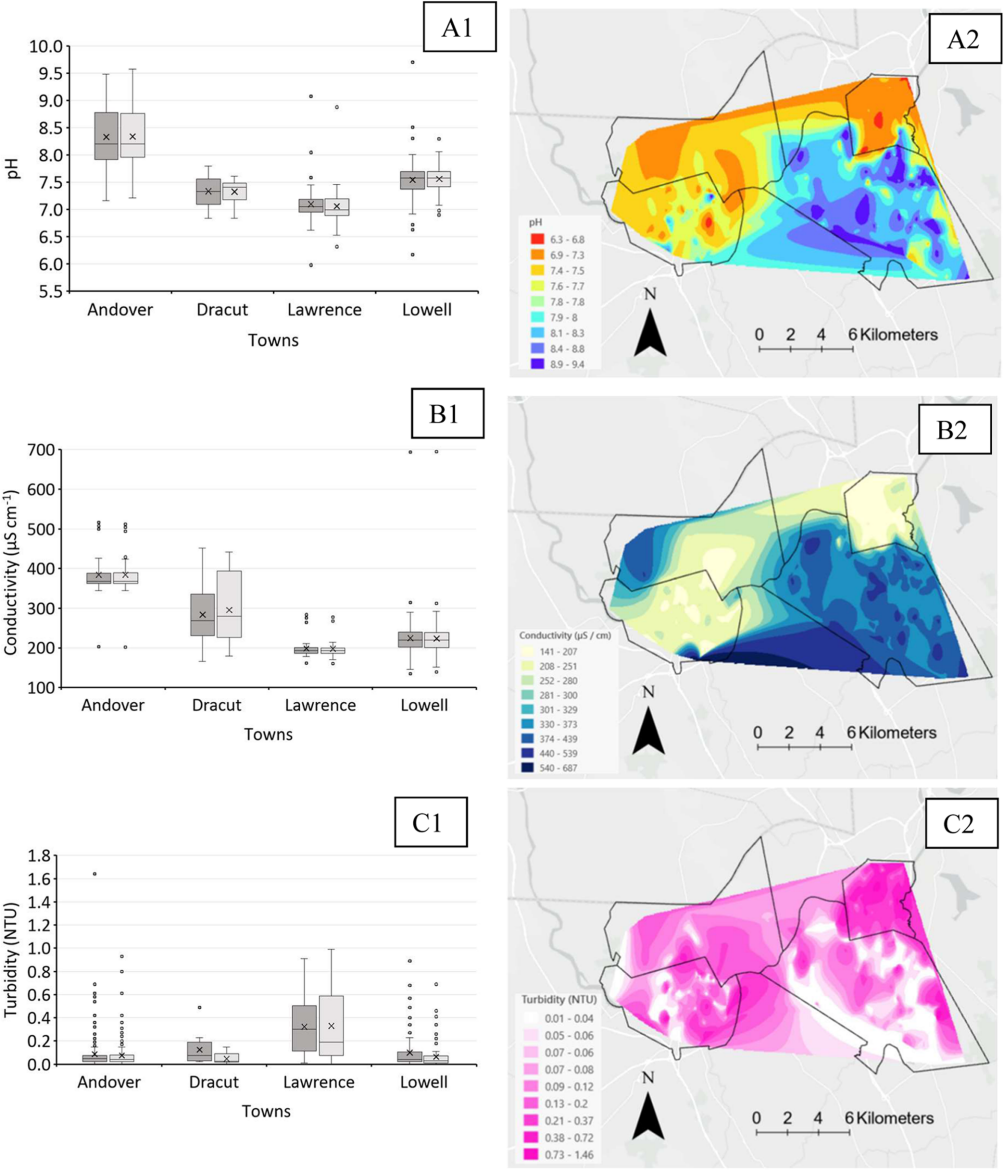
Results of (A1) pH, (B1) conductivity, and (C1) turbidity measurements are shown in box and whisker plots (excluding the median). Dark and light gray bars show first- and fifth-liter results, respectively. X and straight line show the average and median, respectively. The lower and upper parts of the whisker show minimum and maximum values in the interquartile range, respectively. Individual values represent outliers. The pH, conductivity, and turbidity values are interpolated in A2, B2, and C2, respectively.

Water chemistry parameters influence Pb^2+^ leaching from plumbing materials into tap water. To assess community-wide water quality in the target towns, we measured conductivity, pH, and turbidity of all collected samples (Figure 2). As shown in Figure 2A1, tap water pH varies across town. Andover supplies more alkaline water to aid in corrosion control, with pH values for first- and fifth-liter samples ranging from 7.16 to 9.58. In contrast, Dracut provides slightly alkaline water, averaging 7.34 (range: 6.84–7.80). Lawrence supplies the neutral water, with average pH values of 7.10 (first liter) and 7.06 (fifth liter) and a range of 5.98 to 9.08. In Lowell, pH ranges from 6.17 to 9.71, with averages of 7.55 and 7.56 for first and fifth liters, respectively. Figure 2B1 highlights that Andover’s water has the highest average conductivity, followed by Dracut, Lowell, and Lawrence. Notably, Lowell exhibits the widest conductivity range, with values as high as 693.6 μS/cm (first liter) and as low as 134.7 μS/cm (first liter), likely due to its older water distribution system. Lastly, as shown in Figure 2C1, average turbidity is around 0.1 NTU in Andover, Dracut, and Lowell. Only one sample from Andover exceeded 1 NTU (1.64 NTU). Lawrence, however, provides more turbid water compared to the other towns. Spatially interpolated (i.e., natural neighborhood interpolation) results for the first liter measurements of pH, conductivity, and turbidity are shown in Figure 2A2, B2, and C2.

### 3.2 Detection of Lead and Copper in Tap Water Samples by Standard Methods

**Figure 3:**
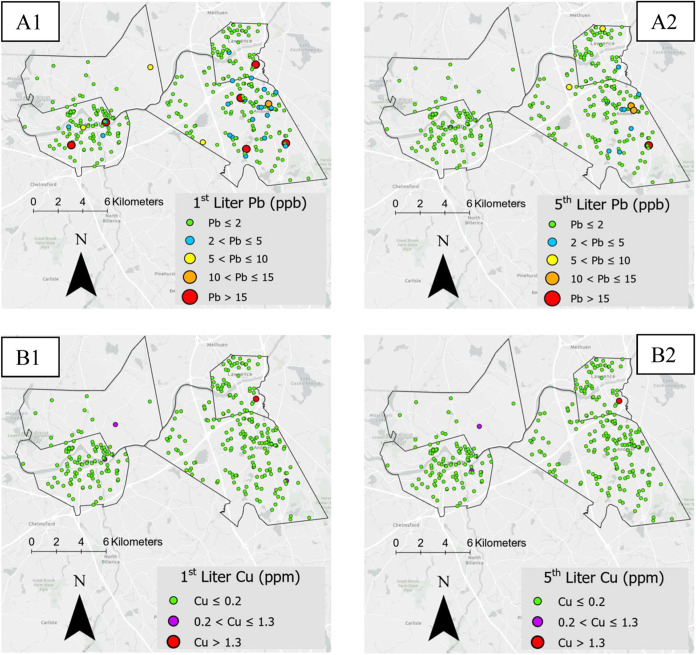
Lead (Pb^2+^) and copper (Cu^2+^) concentrations by color across geographic sampling locations. A1, A2 show first- and fifth-liter Pb^2+^ results, respectively, while B1, B2 display first- and fifth-liter Cu^2+^ results.

The following results detail the concentrations of Pb^2+^ and Cu^2+^ in tap water samples as determined by established standard methods, EPA Method 200.8 (ICP-MS) and EPA Method 200.9 (AAS). These results serve as a reference point for evaluating the performance of the E-Tongue. The first liter of tap water typically exhibits the highest lead concentration, while the fifth liter provides insights into the presence of potential lead service lines.^14,45^ Analysis of the first-liter samples revealed that seven samples (4 from Andover, 2 from Lowell, and 1 from Lawrence) had Pb^2+^ lead concentrations exceeding the old EPA action level of 10 ppb Pb^2+^. Furthermore, three samples had Pb^2+^ concentrations between 5–10 ppb Pb^2+^ and 21 samples were between 2–5 ppb Pb^2+^. All remaining first-liter samples contained less than 2 ppb Pb (Figure 3A1). For the fifth-liter samples, three samples in Andover were above the Pb action level, two samples had Pb^2+^ concentrations between 5 and 10 ppb Pb, and 10 samples ranged between 2 and 5 ppb Pb. All other fifth-liter samples contained less than 2 ppb Pb (Figure 3A2). For Cu^2+^, the highest concentrations from the first- and fifth-liter samples were 14.54 and 2.96 ppm, respectively, both observed in Lawrence (Figure 3B1,B2). These samples were collected from a school laboratory sink, which is not intended to be used for drinking water use. The next highest Cu^2+^ concentrations in the first-liter samples were 0.87, 0.24, and 0.21 ppm, while the corresponding fifth-liter results were 0.82, 0.24, and 0.13 ppm.

Table 1 compares the water quality reported by Andover Drinking Water Treatment Plant (DWTP) outlet and the measured values in collected sample Batch 12 Sample 1 (abbreviated B12 S1), which contained 168.2 and 25.1 ppb of lead in the first and fifth liter, respectively. Except for manganese, the measured water quality parameters were very similar to those reported by the treatment plant. While the treatment plant reported a manganese concentration of 53 ppb, the measured concentrations values were 90 ppb in the fifth liter and 451 ppb in the first liter of the analyzed samples.^46^ Previous studies suggest that elevated manganese levels can increase Pb^2+^ leaching into tap water, which likely explains the high Pb^2+^ concentrations in the first and fifth liters of B12 S1, despite the alkaline pH.^47,48^ Several mechanisms can be attributed to this increase, including manganese acting as an electron acceptor during lead oxidation and suspended colloidal manganese acting as a mobile reservoir for Pb^2+^ released from the corrosion scale due to manganese’s affinity for lead and its larger specific surface area.^48^

**Table 1:** Comparison Between Water Quality Parameters Reported by Andover Drinking Water Treatment Plant (DWTP) and Measured Values of Sample B12 S1 from Andover

parameters	1st liter	5th liter	Andover DWTP outlet^46^
Pb (ppb)	168.2	25.1	not reported
Cu (ppm)	0.87	0.127	not reported
pH	8.06	8.36	8.88
turbidity (NTU)	1.64	0.15	0.23
calcium (ppm)	9.6	9.5	11.3
magnesium (ppm)	1.4	2	2.48
manganese (ppb)	451	90	53
potassium (ppm)	2.8	1.8	2.75
sodium (ppm)	62.9	57.8	59.4
hardness (mg CaCO_3_/L)	30	32	38.5
chloride (ppm)	83.2	83	83
fluoride (ppm)	0.68	0.68	0.76
nitrate as N (ppm)	<0.05	<0.05	0.243
sulfate (ppm)	23.1	23.4	27.1

### 3.3 Voltammetric Detection of Lead (Pb^2+^)

Voltametric experiments were performed using a buffer solution of 1 M sodium acetate and 1 mM potassium ferrocyanide with a pH of 4.5. Potassium ferrocyanide was included in the buffer solution to prevent Cu^2+^ from interfering with Pb^2+^ detection. A common challenge in Pb^2+^ detection is the coexistence of Cu^2+^ in tap water samples.^49^ Since Cu^2+^ is reduced at a more positive potential than Pb^2+^, it can reduce the amount of Pb^2+^ deposited on the electrode surface. In addition, the Pb^2+^ oxidation signal can overlap with the Cu^2+^ oxidation signal, leading to false negative results. Cu^2+^ and Pb^2+^ may also form intermetallic compounds, decreasing the Pb^2+^ signal.^50^ Cu^2+^ interference can be eliminated using complexing reagents.^50−53^ Among various reagents, potassium ferrocyanide was the most effective in eliminating Cu^2+^ interference without significantly affecting the Pb^2+^ signal.^50^ A submillimolar concentration of ferrocyanide can effectively eliminate the interference of a relatively high concentration of copper. It has been attributed to the formation of a less soluble copper complex with ferrocyanide in the solution.^50^ It was found that 0.1 mM of potassium ferrocyanide was sufficient to eliminate the interference of copper with a concentration of Cu^2+^ up to 1.3 ppm.

**Figure 4:**
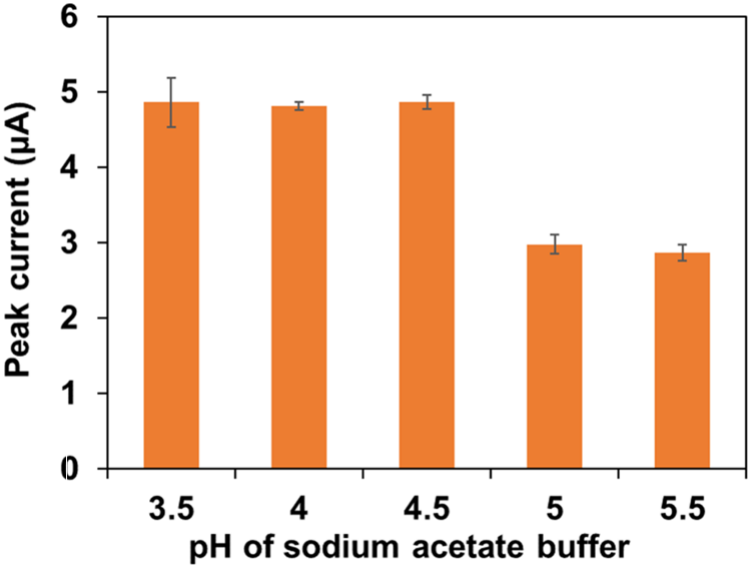
Changes in peak currents for the detection of 25 ppb Pb in buffer solutions of different pH values from 3.5 to 5.5.

Optimization of the pH of the sodium acetate buffer solution was essential to ensure the efficient deposition and dissolution of Pb^2+^ on the AuNS/SPCEs. To determine the optimal pH, the detection of 25 ppb Pb was carried out at pH values ranging from 3.5 to 5.5. Peak currents were high and consistent at pH values from 3.5 to 4.5 but decreased significantly at pH values above 4.5 (Figure 4). The highest peak current with good reproducibility was obtained at pH 4.5, which was selected as the optimum buffer pH for detecting Pb^2+^. The decrease in the peak current at pH values lower than 4.5 could be due to competition between protons and metal ions for the binding sites during the reductive deposition on the electrode surface. At pH values above 4.5, the decrease in the peak current might be due to the hydrolysis of Pb^2+^ to Pb(OH)^+^.^54^ Numerous previous studies have similarly reported sensitive and selective electrochemical detection of Pb^2+^ using sodium acetate buffer solution at pH values around 4.5, further supporting our observation.^32,55,56^

**Figure 5:**
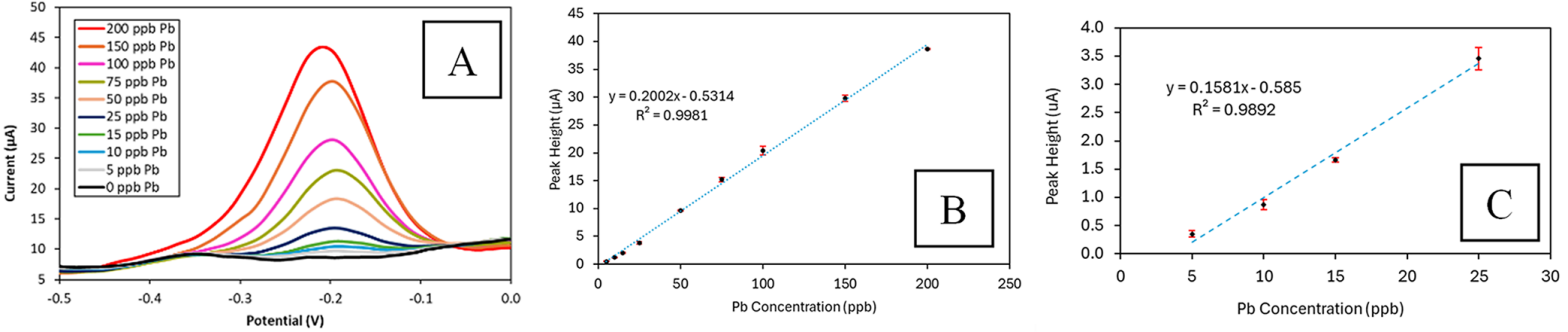
(A) SWASV voltammograms for Pb^2+^ detection at concentrations ranging from 5 to 200 ppb in acidified tap water. (B) Calibration curve from 5 to 200 ppb, (C) calibration curve from 5 to 25 ppb showing peak currents from the voltammograms, with error bars indicating standard deviations from three measurements using three electrodes.

Lead in tap water can exist in two forms: dissolved and particulate.^57^ To investigate this, one of the collected tap water samples was split into two portions to check the presence of particulate lead. One portion was directly acidified, while the other was filtered through a 0.45 μm filter before acidification. The unfiltered sample contained 12.4 ppb Pb^2+^, whereas the filtered sample had less than 2 ppb Pb^2+^ (Table S1). This shows that the collected samples may contain particulate lead. Particulate lead is not electroactive and cannot be quantified by voltammetric methods.^58^ Therefore, calibration curves were developed using acidified tap water (i.e., B42 S3) to ensure detection of total Pb^2+^ in tap water samples. B42 S3 was selected as it contained no detectable lead and represented an average sample with water quality characteristics close to those with more than 10 ppb Pb^2+^. The water quality parameters of B42 S3 are shown in Table S2. Additionally, E-Tongue calibration curves generated using acidified DI water often underpredict lead levels in real tap water samples (Figure S2). Figure 5A–C show the voltammograms for the detection of Pb^2+^at various concentrations in acidified tap water and the corresponding calibration curves. A clear increase in peak current was observed with increasing concentrations of Pb^2+^. The authors found that the data exhibited linear behavior over both the 5–25 ppb and 5–200 ppb concentration ranges. However, a separate linear fit for the lower range of 5–25 ppb showed a stronger correlation, providing greater accuracy near the EPA Action Level of 10 ppb. For this reason, the authors presented a more precise calibration equation specifically for this narrower range (Figure 5C). This methodology had a method detection limit (MDL) of 1.6 ppb Pb^2+^ (Table S3, eq S1 and Figure S3).

**Figure 6:**
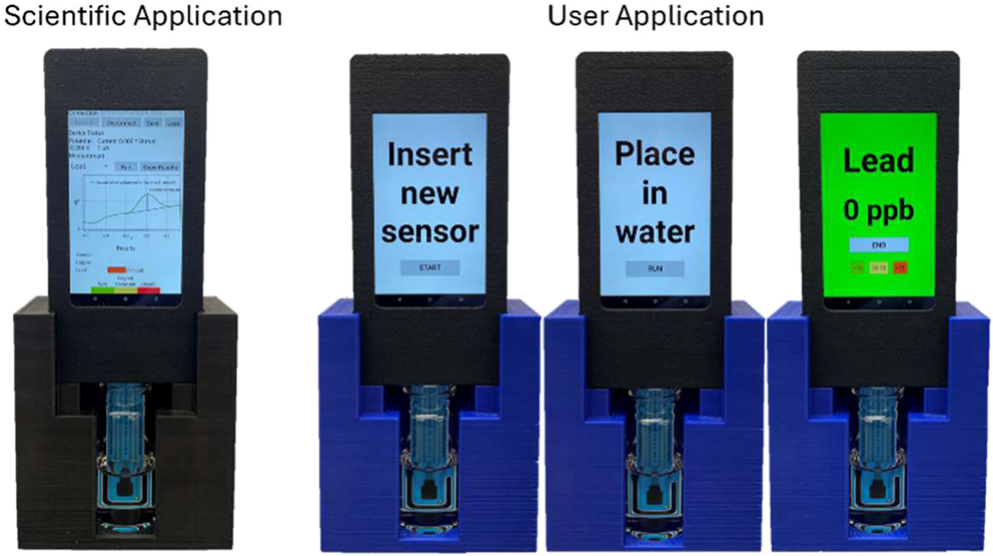
Android applications user interface of E-Tongue for Pb^2+^ detection.

In the developed Android applications for the hand-held device, an algorithm was programmed for peak detection and concentration prediction. The algorithm first calculates the differential curve (the difference between currents measured during the forward and reverse scans). Then, it applies a smoothing filter (i.e., Savitz-Golay filter with a window of 15 data points) to the differential curve. The algorithm subsequently selects a peak around −0.2 ± 0.05 V. Finally, the calibration curve is used to predict the concentration based on the measured peak height. The results table is populated with the predicted concentration and corresponding color code in both the scientific application and user application. In the scientific application, the analyte (e.g., lead) is selected as shown in Figure 6. After clicking “Run”, the experiment is completed in about 5 min, and results are viewed by selecting “Show Results” in the results table. The user application is designed to be more intuitive and straightforward, requiring only three steps: inserting the sensor and clicking “Start”, placing the sensor in the prepared tap water sample and clicking “Run”, and viewing the color-coded, quantitative results. Upon completion, clicking “End” saves the data and returns to the initial screen.

Tap water samples with Pb^2+^ concentrations above the EPA action level (i.e., according to LCRI) were analyzed using the E-Tongue and the developed application. The resulting voltammograms are shown in Figure S4. Table 2 compares the E-Tongue’s performance in acidified tap water samples to AAS. The E-Tongue predicted Pb^2+^ with recovery ranging from 84% to 105% and a relative standard deviation below 10%. Variations in recovery percentage between the samples can be attributed to the differences in complex tap water matrices, including pH, alkalinity, and conductivity.^20,35,59−61^ Nevertheless, the E-Tongue reliably detects Pb^2+^ in drinking water above 10 ppb level within 5 min, significantly faster than the standard methods.

**Table 2:** Results of Pb^2+^ Predictions by E-Tongue and AAS

sample	city	AAS Pb Results (ppb)	E-Tongue Pb prediction (ppb)	E-Tongue recovery (%)	RSD^a^ (%)
B12 S1 1st L	Andover	168.2	155.9	93	9
B51 S1 1st L	Lowell	54.4	52.0	95	4
B7 S6 1st L	Lowell	27.7	23.2	84	1
B12 S1 5th L	Andover	25.1	25.5	102	8
B32 S6 1st L	Andover	19.1	20.0	105	1
B27 S5 1st L	Andover	16.6	15.4	93	2
B35 S1 1st L	Andover	15.6	15.4	99	4
B35 S1 5th L	Andover	15.2	15.9	105	3
B37 S5 5th L	Andover	12.0	11.6	96	4

a RSD is the relative standard deviation.

Several studies have focused on voltammetric methods for lead detection using gold nanoparticle-modified screen-printed carbon electrodes. Table 3 summarizes the performance metrics of these studies, highlighting key performance metrics, including detection limits, linear dynamic ranges, recoveries in tap water, and relative standard deviations. Among the reported studies, the lowest limit of detection (LOD) was achieved by electrode modification B, with an LOD of 2.1 ppt,^33^ followed closely by electrode modification C, with an LOD of 27 ppt.^62^ However, the lowest detectable concentrations using modification B and modification C were 10 and 1 ppb, respectively. Furthermore, modification C involves stirring during the deposition period, making the method significantly less practical for onsite testing. Finally, these methods did not report recovery or precision data in tap water, which limits the assessment of their performance in real sample matrices. Modification D demonstrated a relatively high LOD of 8 ppb and a linear range starting at 200 ppb, suggesting its application may be more suitable for highly contaminated samples. Modification E reported a moderate LOD of 4.2 ppb and a broad linear range (10–270 ppb). However, its recovery ranged between 122 and 138%, indicating possible matrix effects or overestimation in spiked tap water samples. This study (Modification F) achieved a method detection limit (MDL) of 1.6 ppb, an experimentally calculated value unlike other studies based on theoretical LOD calculation. It offers a wide linear range (5–200 ppb), suitable for low-level detection and elevated concentrations. Notably, recoveries in tap water ranged from 84 to 105%, indicating good accuracy, while RSD values ranged from 1 to 9%, demonstrating acceptable precision under realistic conditions. This study provides a balanced performance regarding sensitivity, linearity, and reliability in real water matrices, supporting its applicability for field-based lead monitoring.

**Table 3:** Comparison of Pb^2+^ Detection Studies that Use Gold Nanoparticles Modified Screen-Printed Carbon Electrodes in Tap Water Samples

references	electrode	detection limits (LOD or MDL)^a^	linear dynamic range (ppb)	recoveries in tap water	RSD^b^ in tap water
(21)	A	1.72 ppb (LOD)	1–52	92–96%	1.8–3.6%
(33)	B	2.1 ppt (LOD)	10–100	na^c^	na^c^
(62)	C	27 ppt (LOD)	1–150	na^c^	na^c^
(63)	D	8 ppb (LOD)	200–1000	na^c^	na^c^
(10)	E	4.2 ppb (LOD)	10–270	122–138%	na^c^
this study	F	1.6 ppb (MDL)	5–200	84–105%	1–9%

a LOD = Limit of Detection, MDL = Method Detection Limit.

b RSD = Relative Standard Deviation.

c NA = Not available.

A = Conductive gold nanoparticles and metal complex nanohybrid modified screen printed carbon electrode.

B = Disposable screen-printed carbon arrays modified with gold nanoparticles.

C = Carbon electrode modified with a bismuth film and gold nanoparticles.

D = thiolated calix[4]arene derivative modified on gold nanoparticles and a screen-printed carbon electrode.

E = Screen-printed carbon electrode modified by fluorescent carbon dots and gold nanoparticles.

F = Gold nanostar modified screen printed carbon electrode.

## 4 Conclusions

This study evaluated the performance of a voltammetric sensor for on-site detection and monitoring of lead in drinking water distribution systems. Tap water samples were collected from four Massachusetts communities—Andover, Lawrence, Dracut, and Lowell—and analyzed for lead and copper levels using EPA standard methods. Additional water quality parameters, including pH, conductivity, and turbidity, were also measured, with results mapped to geographic sampling locations. The findings revealed significant variations in water chemistry and metal contamination across the communities. For instance, Andover provided more alkaline water (average pH 8.33), while Lawrence had neutral water (average pH 7.1). Andover also exhibited the highest water conductivity, whereas Lawrence had the lowest. Turbidity measurements showed that Lawrence had the largest interquartile range, while Andover had the smallest. Notably, 10 samples exceeded the EPA lead action level including first- and fifth-liter samples.

This study demonstrated the effectiveness of the E-Tongue as a rapid and reliable tool for detecting lead in drinking water. The hand-held device and an Android-operated application system provides Pb^2+^ detection results in approximately 5 min, with a wide linear dynamic range of 5 to 200 ppb Pb and an MDL of 1.6 ppb. Furthermore, the method reliably detects Pb^2+^ concentrations above 10 ppb, even in the presence of high Cu^2+^ levels of up to 1.3 ppm. Tap water samples with lead concentrations exceeding the EPA Action Levels were analyzed using the E-Tongue, and the results were validated against standard methods. The E-Tongue achieved Pb^2+^ recovery ranging from 84 to 105%, with a relative standard deviation below 10%. While slight underprediction of lead concentrations was observed in some samples—likely due to chemical interferences—the device reliably detected Pb^2+^ levels above 10 ppb within 5 min, significantly faster than conventional EPA methods. In addition to its speed and accuracy, the E-Tongue provides quantitative and color-coded feedback that enables nonexperts to identify Pb^2+^ levels relative to EPA thresholds. Its portability and user-friendly interface make it a practical alternative for community-driven monitoring of lead contamination. By empowering individuals to test and share water quality data, the E-Tongue facilitates the early detection and mitigation of lead exposure risks in water distribution systems. Furthermore, this study demonstrated the potential of integrating Geographic Information Systems (GIS) with portable sensors for spatial analysis of water quality. The combination of real-time, in situ testing with GIS-based trend identification enhances the ability to monitor drinking water quality across different locations. Additionally, the capability to upload E-Tongue data to a cloud environment enables the development of advanced statistical and machine learning models for selecting sampling locations and identifying lead service lines in water networks. This integrated approach offers a scalable solution to improving water quality management and addressing lead contamination more efficiently.

## Supporting Information Available

Additional data on UV–vis spectroscopy to characterize the gold nanostar solution, testing for particulate and dissolved lead, voltammetric calibration curves, method detection limit, and voltammograms for each drinking water sample (PDF)
