# Supplementary material for: Community-Wide Monitoring of Lead in Drinking Water Distribution Systems Using Hand-Held Voltammetric Sensors and Geographic Information Systems
Source: ACS Omega. 2025 May 1;10(18):19096–106. doi: 10.1021/acsomega.5c01580 (PMC12079195; doi:10.1021/acsomega.5c01580)
Supplement: Supplementary file 1 — ao5c01580_si_001.pdf [file ao5c01580_si_001.pdf]

SUPPORTING INFORMATION

for

Community-Wide Monitoring of Lead in Drinking Water Distribution Systems using Handheld Voltammetric Sensors and Geographic Information Systems

Yigit C. Bozkurt[1], Al-Monsur Jiaul Haque[1], Connor Sullivan[1], Boyang Xiang[1], Yidong Zhu[2], Mohammad Arif Ul Alam[2], Pradeep U. Kurup[1]*

[1]*Department of Civil and Environmental Engineering, University of Massachusetts Lowell, Lowell, MA 01854, United States*

[2]*Department of Miner School of Computer & Information Sciencs, University of Massachusetts Lowell, Lowell, MA 01854*

February 2025

*Corresponding author: email: pradeep_kurup@uml.edu; phone: +1 978-934-2278

UV-VIS absorption spectra:

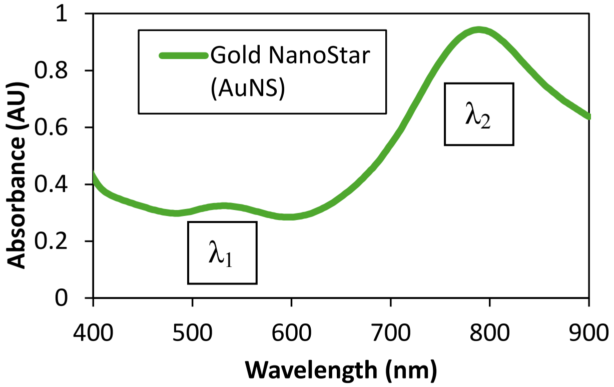

Figure S1 UV-VIS absorption spectra of the synthesized gold nanostars

Particulate and Dissolved Lead:

Table S1 Comparison of the Pb^{2+} detection in unfiltered vs. filtered samples by GF-AAS

**Sample**	**Pb Concentration by GF-AAS (ppb)**
Unfiltered B32 S6 1st Liter	12.4
Filtered B32 S6 1st Liter	0.6

Table S2 Water of quality of Batch 42 Sample 3

Sample	pH	Conductivity (µS/cm)	Turbidity (NTU)
B42 S3 1st	8.54	350.9	0.58
B42 S3 5th	8.62	346.8	0.93

Pb^{2+} Calibration Curves:

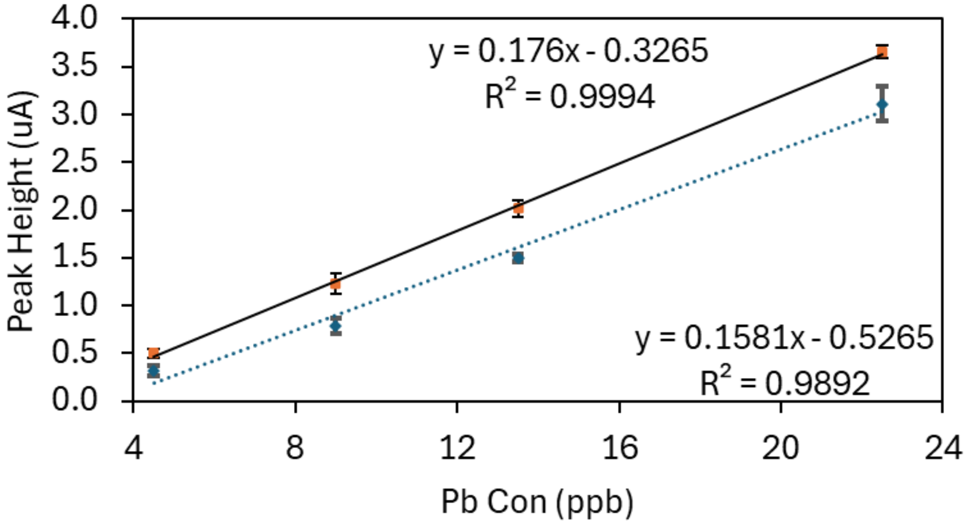

Figure S2 Spiked lead nitrate into acidified DI (orange data points) vs acidified tap water (blue data points)

Method Detection Limit:

The method detection limit (MDL) was calculated below.

$$MDL = t\ x\ S\ (\boldsymbol{S1})$$

Where:

- t is the Student's t value for a 99% confidence level and a standard deviation estimate with n-1 degrees of freedom [t = 3.14 for seven replicates]
- S is the standard deviation of the replicate analyses

Table S3 Seven replicates of 5 ppb Pb^{2+} detection by E-Tongue

Sample ID	Peak Height (μA)	E-Tongue Pb Prediction (ppb)
5 ppb Pb	0.359	6.2
5 ppb Pb	0.358	6.2
5 ppb Pb	0.517	7.3
5 ppb Pb	0.397	6.5
5 ppb Pb	0.504	7.2
5 ppb Pb	0.511	7.3
5 ppb Pb	0.495	7.2

The standard deviation of seven replicas is 0.5188 ppb Pb

$$MDL = 3.14\ x\ 0.5188\ (\boldsymbol{S1})$$

$$MDL = 1.6\ ppb\ Pb^{2+}\ (\boldsymbol{S1})$$

The seven replicates at 5 ppb were performed specifically to determine the method detection limit. This limit is below the EPA action level of 10 ppb. At the lower concentration range around 5 ppb, the E-Tongue can overpredict by 1 to 2 ppb, which the authors believe is acceptable for a field screening device.

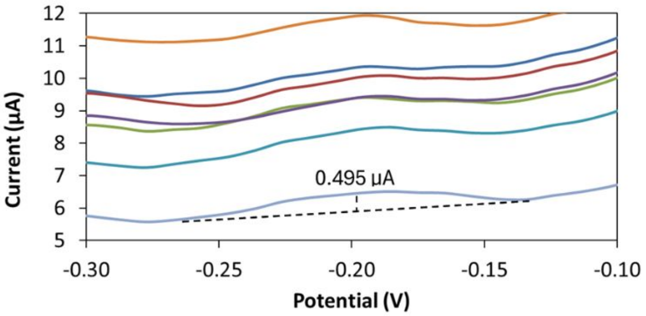

Figure S3 Seven replicates of 5 ppb Pb^{2+} voltammograms

Voltammograms in Drinking Water Samples:

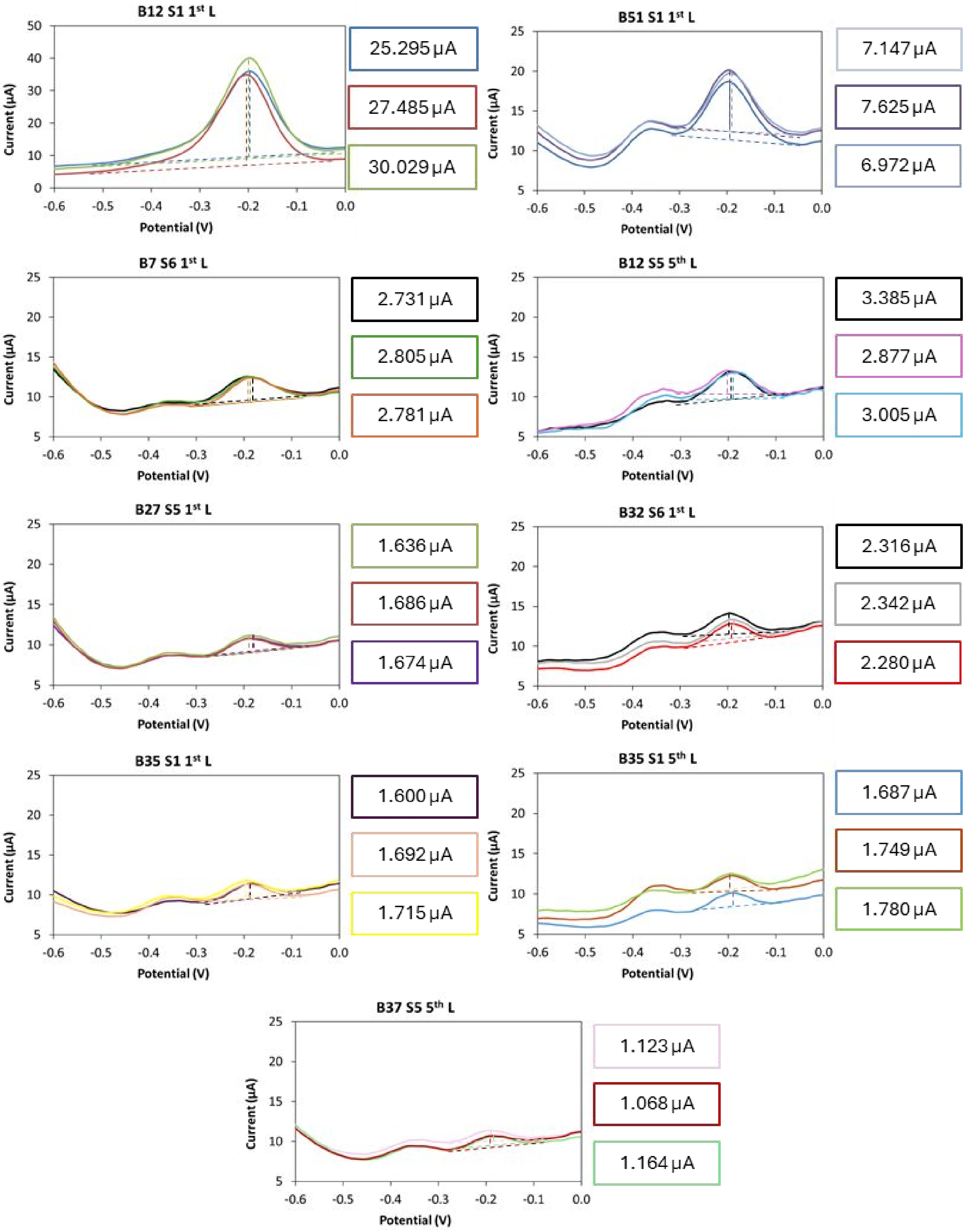

Figure S4 Voltammograms for the detection of Pb^{2+} in the samples, B12 S1, B51 S1, B7 S6, B32 S6, B27 S4, B35 S1, and B37 S5.

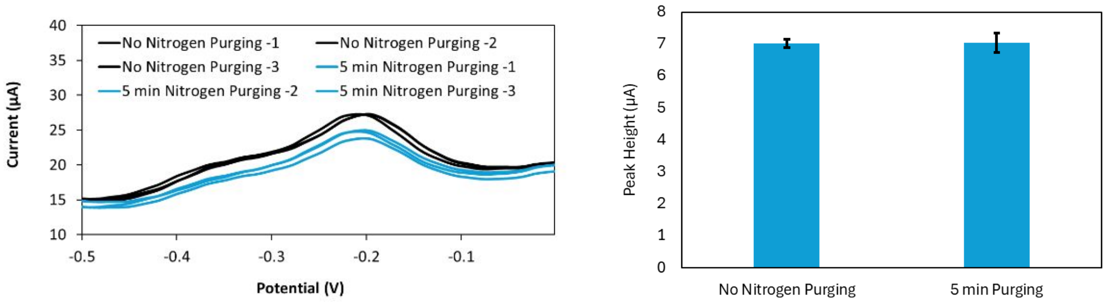

Figure S5 50 ppb Pb detection with and without nitrogen purging
